# Supplementary material for: Land-use intensity of electricity production and tomorrow’s energy landscape
Source: PLoS One. 2022 Jul 6;17(7):e0270155. doi: 10.1371/journal.pone.0270155 (PMC9258890; doi:10.1371/journal.pone.0270155)
Supplement: S1 Text — (DOCX) [file pone.0270155.s001.docx]

**Land use intensity of electricity production and tomorrow’s energy landscape**

Jessica Lovering, Marian Swain, Linus Blomqvist, Rebecca R. Hernandez

**Supplementary Information**

## **Data & Methods**

Below we provide more details on how land use and electricity generation data was collected for each generation source. A summary of all the data sources for each electricity source is provided in Supplementary Table S1.

#### **Coal**

Total LUIE for coal (n = 30) includes direct land impacts from power plant infrastructure and indirect impacts from coal mining, processing, and transportation. Six sources provided data on both direct and indirect LUI [1–6]; two sources only provided indirect LUIE results. Coal data are from the United States and Canada [7, 8].

Fthenakis & Kim (2009) provided the most detailed analysis of coal LUIE [1]. For direct land impacts, they assessed power plants ranging from 500 to 1000 MW and assumed an 85% capacity factor. They included area occupied by the powerhouse, switchyard, stacks, precipitators, walkways, coal storage, and cooling towers, but excluded waste storage areas. For indirect impacts, they assessed the land transformation of both surface and underground mining for 14 regional case studies in the US.

Hertwich et al. (2015) conducted a life-cycle analysis for three different coal power plant types: subcritical, supercritical, and integrated gasification combined cycle [2]. Each was also assessed both with and without carbon capture and storage (CCS) technology (inclusion of CCS reduces efficiency). They assumed that coal is transported by rail over 330 km to the power plant and assessed North American coal mining.

Spitzley & Keoleian (2005) assessed the direct land impacts of three different coal plant types: direct-fire coal boiler, direct-fire with low NOx burners, and U-fired coal boiler with low emissions [3]. For indirect impacts, they assessed surface coal mining in Illinois and assumed a coal transportation distance of 1334 km (an average figure for the northeastern US). Jacobson (2009) cited Spitzley & Keoleian’s figures for both direct and indirect impacts, but under two different capacity factor assumptions (65% and 85%) [4].

Smil (2010) presented data on the Robert W. Scherer coal plant in Georgia, USA, with a 3.5 GW installed capacity and 75% capacity factor [5]. The direct land impacts include ash ponds and storage areas and indirect impacts are from surface mining.

Gates (1985) analyzed US coal plants including area for coal delivery, handling and storage, land for the powerhouse and cooling towers, ash and sludge ponds, roads, parking, switchyards, and landscaping. For indirect impacts, Gates assessed land impacts of underground mining, surface mining, and rail transport [6].

Two studies provided numbers only for indirect LUIE, so we combined these with the average direct LUIE data from the other studies in order to estimate total LUIE [7, 8]. Jordaan (2010) surveyed existing coal mines in Alberta, Canada using publicly available records, including only mine area, not transportation or waste disposal area. Jordaan’s results were for embodied energy, not final electricity, so we applied a conversion efficiency of 35% based on Ftheankis & Kim [1]. McDonald et al. provided a most and least compact estimate for US coal mining [8]. The most compact estimate was from Spitzley & Keoleian [3], while the least compact result was for the Flattop surface mine in the US.

#### **Natural Gas**

Total LUIE for natural gas (n = 17) includes direct impacts from power plant infrastructure and indirect impacts from natural gas drilling and transportation infrastructure. We present footprint (n = 17) and spacing (n = 4) results for indirect LUIE. Footprint LUIE represents the area covered by gas well pads, access roads, and pipelines. Five sources provided data on footprint LUIE [3, 7, 9–11]. Spacing LUIE refers to the entire production field, including all the area in between well pads, even if that land does not have any structures or roads covering it. Two studies provided data on natural gas spacing area and one source [5] provided data only on direct LUIE [8, 12].

The US National Energy Technology Laboratory (NETL) produced a detailed life-cycle analysis of natural gas generation [10]. For direct impacts, they assessed a 360 MW single-cycle power plant and a 565 MW combined cycle plant. For indirect impacts, they assessed conventional and unconventional (tight and shale) gas extraction.

A 1983 US Department of Energy (DOE) document that provided a detailed breakdown of land impacts for natural gas production. For direct impacts, DOE assessed an 800 MW power plant with a 55% capacity factor and indirect area impacts represented typical US natural gas extraction, purification, and pipeline transmission.[9]

Spitzley & Keoleian (2005) assessed a 505 MW combined cycle natural gas plant, including the direct footprint of the power plant and indirect land impact from the pipeline area [3]. They did not include area required for natural gas drilling because they assumed land area requirements of natural gas wellheads to be negligible compared to power plant area.

Smil only provided figures for direct LUIE, based on three different power plant types: the 25 MW PW-brand MobilePac mobile gas turbine, the 60 MW PW-brand SwiftPac mobile turbine, and a 100 MW stationary power plant [5].
 Jordaan (2010) and Bryce (2011) provided figures only for indirect impacts from natural gas drilling. Jordaan (2010) took satellite measurements of medium-size natural gas wells in Alberta, Canada [7]. Bryce assessed average US natural gas wells and gas stripper wells assuming 33% efficiency [11].

McDonald et al. and Copeland et al. are the only two studies we identified that provided calculations for indirect spacing LUIE. McDonald et al. (2008) assessed the area fragmented by natural gas drilling and pipelines, taking a most and least compact measurement from US production practices [8]. Copeland et al. took data from the US Bureau of Land Management (BLM) for United States natural gas leases and spatially analyzed active Canadian leases using a geographic information system [12]. They assess the area covered by well pads, access roads, and pipelines.

#### **Nuclear**

Land use for nuclear includes direct impacts from the power plant and indirect impacts from the uranium fuel cycle, including mining, milling, conversion, enrichment, and fabrication. Our dataset covers all operating nuclear power plants in the United States (n = 65) as of 2014. We chose the United States since it has a large fleet of active nuclear power plants representing a range of technologies. EIA provides data on each plant’s electricity output; we used data from 2014 [13]. We combined these data with area measurements of the footprint (power plant infrastructure) using satellite imagery.

For the footprint area, we made area measurements in Google Earth Pro. We located each power plant on a satellite map and traced a polygon around its perimeter to determine the site area. We defined the footprint area as reactor buildings, cooling towers, cooling ponds (if applicable), dry cask waste storage, administrative buildings, parking lots, and any other human structures that are associated with the plant’s operation.

Environmental Impact Statements from the Nuclear Regulatory Commission also provided land area data for most US nuclear plants (n = 60) [14]. These reports include a measurement of “total site area,” which includes a variety of non-essential structures and ranges from 1 to 250 times greater than our measurement of power plant area. Many sites include additional unoccupied land that was set aside for future reactors that were then cancelled, while other power plants have set aside buffer zones to reduce the number of people living in their emergency planning zone for potential accidents. Calculating LUIE for nuclear using these expanded areas results in a median value of 49 ha/TWh/y.

For indirect LUIE for nuclear, we acquired uranium mining and processing land area data from several literature sources [1, 13, 15–19], covering mining activity in Australia, Canada, Namibia and the U.S. There is only one study we found that provides an estimate for the other aspects of the front end of the nuclear fuel cycle: conversion, enrichment, and fabrication [1]. We use these figures in our aggregate, but they may not be representative as the U.S. has mostly exited the nuclear fuel cycle. All of these studies provide measurements of the area required to produce a certain amount of uranium fuel; we thus had to convert into a per unit of electricity figure to add to the direct LUIE for power plants.

Regarding spent nuclear fuel or nuclear waste: in the US, spent fuel is stored on-site and is therefore included in our direct LUIE calculation. However, two studies estimated the land occupied by the now-cancelled Yucca Mountain waste repository in the U.S. [1, 20]. Fthenakis & Kim (2009) relied on the entire land withdrawal of 150,000 acres for Yucca Mountain, which we estimate as a LUIE of 2.9 ha/TWh given the amount of spent fuel Yucca could have contained and how much electricity was produced from that fuel. Jacobson (2009) uses a narrower definition of land impacted by infrastructure of Yucca Mountain and finds a range of LUIE from 0.0093-0.0139 ha/TWh. Including this land in nuclear’s total LUIE does not significantly alter the result, but it is also purely hypothetical at this point as the U.S. does not have a long-term solution for nuclear waste. Other countries like France and Japan recycle their spent fuel which result in a much smaller footprint of waste to be stored.

One unique type of land impact associated with nuclear are exclusion zones created around nuclear power plants after an accident. The two major accidents in nuclear power’s history were at the Chernobyl plant in Pripyat, Ukraine, which resulted in an exclusion zone of 260,000 ha [21], and at the Fukushima Daiichi plant in Japan, which resulted in an exclusion zone of 63,000 ha [22]. We calculated the LUIE of nuclear accidents by combining these two exclusion zones and dividing that area by total historical nuclear power generation (~82,000 TWh) [23], which resulted in a LUIE of 3.9 ha/TWh/y. However, in both cases, the exclusion zones are at least partially inhabited and, in the case of Chernobyl, the zone is occupied by abundant wildlife [24]. Such events are difficult to compare across other energy sources and the challenge in measuring these impacts is how to define land occupation and land use.

#### **Hydroelectric**

The direct area of hydroelectric dams is the area flooded by the reservoir. Our dataset (n = 952) is compiled from International Commission on Large Dam’s World Register of Dams database and represents single-use hydroelectric dams in eighty countries [25]. The World Register of Dams provided data on mean annual electricity generated, dam height, age, and reservoir area.

The land impacts of hydroelectric are unique because they represent an area of land that has been intentionally flooded, thereby creating a permanent reservoir. Even though artificially-created reservoirs may serve as functioning habitat area for aquatic and terrestrial species, we include the reservoir as a land use impact since it was created wholly or in-part for the purposes of energy production. Additionally, although about 40% of hydroelectric dams serve multiple purposes beyond power generation [25], in this study we attribute the entire reservoir area to power generation.

We exclude run-of-the-river hydroelectric projects since they represent a small portion (roughly 4%) of worldwide hydroelectric capacity and reliable generation data could not be found [26]. However, results from Fthenakis & Kim (2009) suggest LUIE for run-of-the-river projects are much smaller than for traditional hydroelectric (about 10 ha/TWh/y) [1].

#### **Biomass**

Like other combustibles, the land impacts from biomass include the area of the biomass power plant (direct LUIE) as well as the area needed to supply the feedstock for the plant (indirect LUIE). Our dataset for dedicated biomass (n = 14) represents woody biomass production from willow, poplar, and spruce trees. Data are drawn from six sources.[1, 3, 8, 27–29] For residue biomass, no indirect land area is included because we assume no land requirement for residue feedstock production.

We get direct plant area from Spitzley & Keoleian (2005), who provide generation and area information for four biomass plants with a range in LUIE of 102-230 ha/TWh/y [3]. Coal power plants can also be used as a proxy since it is common to retrofit a coal plant to burn biomass. Our direct LUIE results for coal power plants ranged from 18-98 ha/TWh/y, but we would expect a biomass plant to have a larger LUIE since the plant runs at lower efficiency.

Spitzley & Keoleian provide the most detailed assessment of dedicated biomass [3]. They examined willow biomass grown in New York State and hybrid poplar biomass grown in the Midwestern US. They also calculated LUIE under three biomass power plant technology types: low-pressure gasification, high-pressure gasification, and direct-fire boilers.

Fthenakis & Kim took crop yield data from Spitzley & Keoleian (2005) and another literature source and made LUIE calculations under conditions of the following power plant technology types: low-pressure gasification, high-pressure gasification, direct-fire, and co-firing [1].

Dijkman & Benders (2010) assessed woody biomass production from short rotation coppice systems in Europe under different yield assumptions: lowest European yields in the period 2000-2007, highest yields in that period, and average yields [27].

Smil (2010) looked at woody biomass from fast-growing willow, poplar, and pine plantations. He calculated a land requirement of 330,000 hectares for a 1 GW power plant with a 70% capacity factor and a 35% conversion efficiency [29].

Kumar et al. (2003) provided LUIE data for wood-burning biomass plant in Alberta, Canada [28]. They assessed a 900 MW power plant burning whole forest biomass from mixed hardwood and spruce trees. They took yield data from western Canadian forestry and calculated heat content for the wood feedstock of 20 GJ/ton.

McDonald et al. (2009) included two biomass LUIE calculations: one for willow gasification under existing yield assumptions, and another for assuming higher future yields [8]. Since we are including only real-world results, we excluded the LUIE values for hypothetical improved yields.

#### **Wind**

Land impacts from wind come from the area covered by wind turbines and access roads. We calculate both footprint and spacing LUIE results for wind (n = 57). Footprint area represents only the area physically covered by the turbine pad and access roads; spacing area includes all the area in between turbines. Our dataset is generated from a random sample of operating US wind farms over 20 MW from EIA. We used EIA Form 860 and Form 923 to gather data on installed capacity and annual electricity output for each wind farm for 2013 [30, 31]. We combined this with measurements of the footprint and spacing area of each wind farm calculated using Google Earth Pro. For footprint area, we traced perimeters around each turbine pad and the access roads connecting them. For spacing area, we traced the perimeter of the entire wind farm, including all the space in between turbines.

#### **Solar**

We assessed the LUIE of integrated PV, ground-mounted PV, and solar CSP facilities. Integrated PV, which is placed on existing structures in the built environment, is given a LUIE of 0 ha/TWh/y in this study since it does not have an additional land footprint. Our datasets for ground-mounted PV (n = 94) and CSP (n = 7) are based on existing, operational plants over 20 MW in 18 US states with capacity factors over 5%. For all sites, annual electricity generation data came from EIA Form 923 data for 2014 [31].

To expand the data on CSP, we also included a broader dataset from Lilliestam et al. (2021) that includes land use and expected electricity generation for an additional 76 CSP projects across 13 countries. We included this data even though it is an estimate for generation because we had so few real sites for CSP plants. We also included an additional 20 sites across the U.S. from Ong et al. (2013), who uses two different capacity factors to provide a range of generation for each plant, and we averaged them for our dataset. We removed any duplicates, keeping the plant the LUIE estimate that had real values for electricity generation or was the more recent estimate. For the plants where we can cross-reference real generation from the EIA, we find that the Lilliestam and Ong data overestimate electricity generation by roughly 30% on average. This would bias the LUIE calculation downward for CSP.

Area measurements came from Hernandez et al. (n = 17);[32] Ong et al. (n = 68);[33] Solar Energy Industries Association (n = 12);[34] NARENCO (n = 4);[35] and BLM (n = 3) [36]. For ground-mounted PV and solar CSP, we define direct area as the area of panels or heliostats, roads established during development, and all ancillary facilities. Ancillary facilities may include new service roads, power collection systems, communication cables, overhead and underground transmission lines, electrical sites, switchyards, project substations, meteorological towers, thermal storage units, and operations and maintenance facilities.

#### **Geothermal**

Geothermal land impacts include the area covered by power plant infrastructure and injection wells. Bertani provided a detailed list of worldwide geothermal power plants, their running capacity, the number of production wells, the reservoir temperature, and the drilled area [37]. We calculated annual electricity output from running capacity. The drilled area presented in Bertani, however, represented the entire expanse of the underground geothermal reservoir, only a fraction of which had above-ground land disturbance from the power plant and production wells. We took a sample of 26 facilities listed in Bertani for which annual electricity generation data was provided. We combined this with land use data from geospatial measurements from the Global Energy Observatory (GEO) online database [38]. Our dataset (n = 26) represents 11 countries.

**Regressions**

We conducted regressions to see if LUIE for each source could be predicted by any physical variables for each electricity source, such as plant capacity, specific technology, or location. All results are reported at the 95% confidence level. For natural gas (footprint), a multiple regression with power plant capacity and extraction technology (shale, tight, and conventional) shows that power plant capacity has a significant negative correlation with LUIE (p=0.001), and that shale extraction has significantly lower LUIE than the other two extraction technologies, when accounting for power plant capacity (p=0.043).  For dedicated biomass, LUIE shows a strong negative relationship to feedstock yields (p=0.004), and hardwood and spruce have significantly higher LUIE than poplar or willow (p=0.004).

In a multiple regression for hydroelectric, dam height is positively correlated with LUIE (p<0.005), whereas capacity is negatively correlated (p<0.005). Hydro-only dams have significantly lower LUIE than dams with mixed uses (p<0.005). This is not surprising since the flooded area might be the same, but some of that water will be diverted to, for example, irrigation, which will reduce the amount of electricity generated. A number of countries also show strong correlations with LUIE for hydro. Sweden has the lowest LUIE relative to all other countries, followed by Guinea, Sri Lanka, Slovakia, Peru, and Ecuador. Burkina Faso, Ivory Coast, Tanzania, Zambia, and Zimbabwe all had significantly higher LUIEs than other countries.

**Applying LUIE Results to Global Energy Scenarios**

We applied our mean LUIE results to the electricity mix of future scenarios for the global power sector, as well as to today’s global electricity mix [39], to determine the current and projected land requirements for future global electricity roadmaps. Our LUIE results suggest that current total global land use for electricity production is approximately 72 (±1.7) Mha, with 80% of that land used for hydroelectric dams.

We assessed ten global decarbonization pathways from six different organizations and studies: the 2, 4, and 6 degree Celsius scenarios from the International Energy Agency’s *Energy Technology Perspectives* (hereafter “IEA”) [40], Greenpeace’s *Energy [R]evolution* (“GP”) [41], World Wildlife Fund’s *Energy Report* (“WWF”) [42], three scenarios from the *Global Energy Assessment* (“GEA”) [43], Jacobson & Delucchi (“JD”) [44], and Barry Brook (“Brook”) [45]. Real-world land requirements vary by region and the dynamics of land-use change are highly context-dependent. These projections are not intended as forecasts, but rather as estimates of the scale of land use that would be needed for electricity production in hypothetical decarbonized electricity portfolios. Total electricity demand and generation mix for each scenario is provided in Supplementary Figure S1.


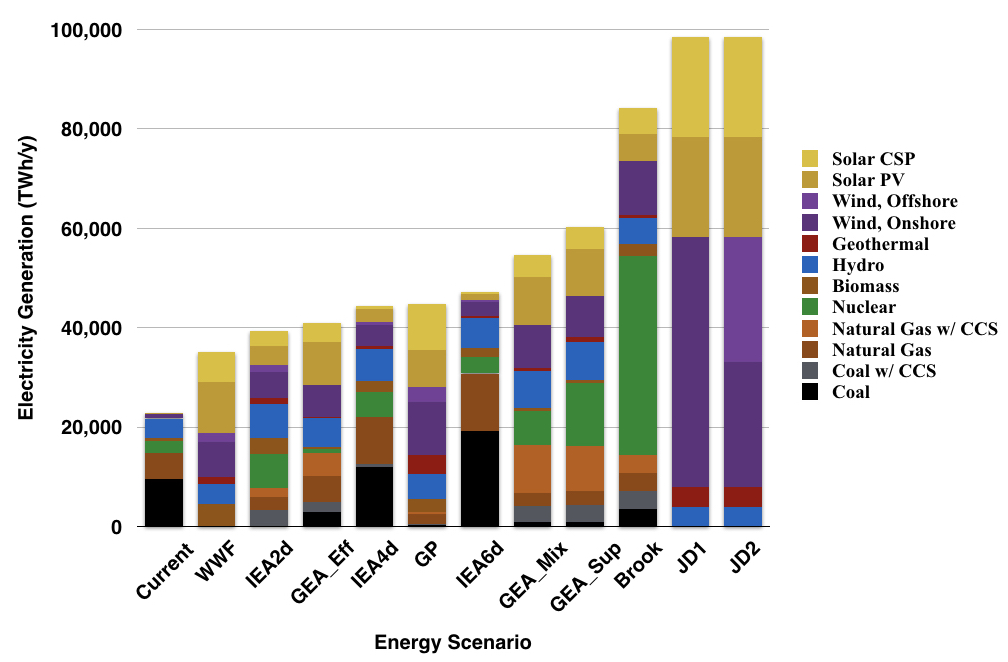


**Supplementary Figure S1.** Electricity generation profile by scenario. All scenarios are for global demand. Natural gas and coal include generation both with and without carbon capture and storage (CCS). The current generation mix comes from EIA 2017 data.

The scenarios reviewed here all have different assumptions about electricity’s share of final consumption. JD envisions a 100% electrified energy sector and Brook assumes 95% electrification as part of a decarbonization strategy. All other scenarios assume less than half of final energy will be from electricity, as is true of the current energy system. Land impacts from non-electric energy are not accounted for here, but would increase the overall land requirements for those scenarios.

Several scenarios include electricity generation from natural gas and coal with carbon capture and storage (CCS) technology, which is not accounted for in our LUIE results. Adding CCS to a thermal power plant decreases efficiency and thus increases LUIE compared to our non-CCS results. To account for this difference, we increased our LUIE result for coal and natural gas by 40% for generation with CCS based on Hertwich et al. (2015) [2]. IEA and GEA scenarios also included biomass with CCS, but since the share of biomass with CCS was less than 1% of total generation, we did not calculate it separately.

JD, GP, and WWF distinguish between onshore and offshore wind in their scenarios. We exclude offshore wind from our analysis and only calculate land requirements for onshore wind. JD distinguish between rooftop and ground-mounted PV: we assume the land impacts of rooftop solar to be zero and calculate the land requirement only for ground-mounted PV. For all other scenarios where rooftop vs. ground-mounted was not specified, we assumed all PV was ground-mounted to represent an upper bound on land requirement. Our scenario results include both footprint and spacing area for wind and natural gas; Supplementary Table S2 shows how much of the overall spacing area required in each scenario is from natural gas and wind. We excluded marginal electricity sources that we did not include in our LUIE analysis (wave and tidal power, hydrogen, and oil) from our land requirement calculations.

The Brook scenario required several additional assumptions. The author provided a combined figure for electricity generation from wind and solar; to make our land requirement calculations, we assumed half of that amount was solar and half was wind. We assumed all wind to be onshore, and that solar generation was split evenly between ground-mounted PV and CSP. The author also provided only a single figure for fossil fuel with CCS, so we assumed half from natural gas and half from coal and adjusted LUIE to account for CCS inefficiency. We assumed all electricity categorized as “other renewables” was from geothermal. Depending on how the wind and solar generation is divided, the overall land requirement (including spacing area) could be 30% higher or lower. Changing the assumptions about coal and natural gas or about solar CSP and ground-mounted PV would create at most a 1% difference in land requirement.

To apply our mean LUIE figures for each electricity source to the global energy scenarios, we multiplied each LUIE by the annual electricity generation for each source and totaled them. The total land area required for each scenario is shown in Supplementary Figure S2. We also propagated the error for each electricity source to provide error bars on the total land use for each scenario.

**
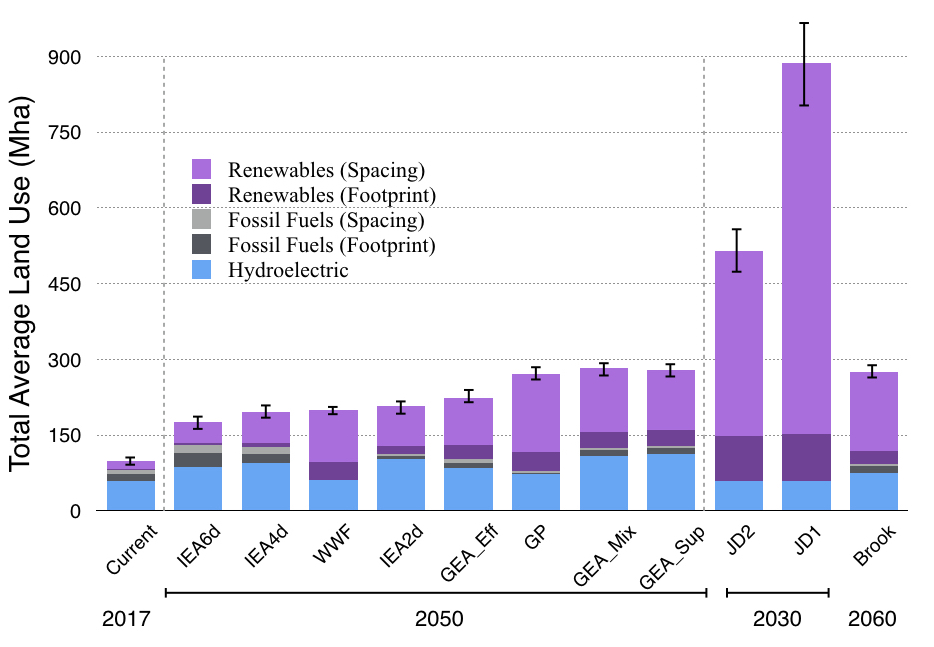
**

| **Supplementary Figure S2.** Land area (Mha) for future electricity generation scenarios, broken down by source of land use: hydroelectric, fossil fuels, non-hydro renewables, and spacing from wind and natural gas. Land use for biomass electricity is included in non-hydro renewables, but we assume all biomass comes from residue or waste for these calculations, thus representing a lower bound. JD1 refers to the Jacobson & Delucchi scenario assuming all wind is onshore, and JD2 assumes 50% of wind is onshore and 50% is offshore. Total land required to generate electricity in each future decarbonization scenario is shown with standard errors. GEA_Sup, GEA_Eff, and GEA_Mix are the GEA Supply, Efficiency, and Mixed scenarios respectively. Electricity generation data for the current mix (2017) comes from the BP Statistical Review. |
| --- |

Our analysis suggests the possibility of a significant expansion of the land footprint for electricity in the coming decades, ranging from an additional 30-80 Mha for physical footprint to and additional 80-800 Mha when spacing is included. The scenario with the lowest total land-use was the IEA 6 Degree scenario, which is a business-as-usual scenario that includes a large share of fossil fuels. The WWF and Greenpeace scenarios also had low total land use, but this was in part due to their lower overall projected electricity consumption, as well as their limited reliance on large hydroelectric. Brook had lower land-use despite higher overall electricity consumption, primarily due to their reliance on nuclear power, which has the lowest LUIE. The Jacobson scenarios had the highest land use both because they were converting all global energy use to electricity, and they also rely extensively on wind and solar.

The projected expansion of land-use across these scenarios is a similar order of magnitude to the value projected for global urban expansion (60-241 Mha) [46], and when spacing is included this may exceed forecasted cropland expansion (average 160-320 Mha of various projections) [47]. If biomass was to come from dedicated feedstocks, the additional land required would be between 80 and 700 Mha across these scenarios. For comparison, Jacobson et al. (2017) estimated that the land required for a 100% renewable system would be lower than our calculation (35 Mha or 177 Mha with spacing), but their land-use figures represent hypothetical electricity generation, which tends to be lower than realized generation from our surveys [48]. Trainor et al (2016) calculated additional land use from EIA scenarios in the US and found land use could grow by 18-24 Mha by 2040, but this is for all energy production in the US (not just electricity) [49].

**Supplementary Table S2.** Spacing area in energy scenarios. Spacing area (Mha) is calculated for wind and natural gas generation. In all scenarios, the majority of spacing area is for wind.

|  | **Spacing area (Mha)** | **Spacing area from natural gas (%)** | **Spacing area from wind (%)** |
| --- | --- | --- | --- |
| **Current (2015)** | 20 | 38% | 62% |
| **IEA 6DS** | 57 | 29% | 71% |
| **IEA 4DS** | 75 | 18% | 82% |
| **IEA 2DS** | 83 | 5% | 95% |
| **WWF** | 100 | 0% | 100% |
| **GEA "Efficiency"** | 100 | 8% | 92% |
| **GEA "Supply"** | 120 | 3% | 97% |
| **GEA "Mix"** | 130 | 3% | 97% |
| **Greenpeace** | 160 | 2% | 98% |
| **Brook** | 160 | 3% | 97% |
| **JD1** | 730 | 0% | 100% |
| **JD2** | 370 | 0% | 100% |

**Uncertainties & Further Research**

Our results cover a sufficiently large number of samples with significant geographical spread to provide broadly representative figures. However, variances are large, suggesting substantial differences in the LUIE of individual energy sources depending on geographical, technological, and other factors. Our numbers thus may not be applicable at scales below regional or national.

Additionally, the GHG emissions intensity of some electricity sources, especially biomass and hydroelectric, are contextual, site-specific, and sometimes disputed [50, 51]. Although biomass electricity is often identified by policy-makers as carbon neutral [52], Searchinger & Heimlich (2015) note that many assessments of carbon emissions from bioenergy do not fully account for GHG emissions associated with land conversion. If natural landscapes are cleared for bioenergy production there is an immediate release of that area’s stored carbon, a carbon debt that can take decades or even centuries to recoup [53].

Several scenarios (GP, WWF, and GEA) specify that the biomass in their scenarios should come only from forestry and agricultural wastes and residues, rather than dedicated production, to minimize negative impacts from land use change and avoid competition with food crops. Those scenarios project 1-16 EJ of final energy consumption from biomass from residues. For comparison, Searchinger & Heimlich (2015) estimate that 19-35 EJ of wood waste and residues are available globally [53]; however, this is the technical potential, not the amount that would be economically recoverable. In a study of Western North America, Sanchez et al. (2015) estimate that 1.86 EJ of biomass from waste and residues could be recovered annually [54]. They estimate that this amount, combined with a small amount of dedicated biomass (0.07 EJ), could meet 7-9% of the region’s modeled electricity demand in 2050.

Our study highlights several areas for further research. New studies could provide better geographic representation, for example by replicating our calculations of nuclear, wind, or solar with data from outside the United States. This applies especially to wind and solar, which rely on natural resources that can vary significantly based on local conditions. For nuclear, regulation affecting plant cooling technology, safety, and security staffing can affect the LUIE of plants in different countries.

**Sourcing of Biomass Carries Significant Uncertainty**

Future biomass demand will likely be met by a mixture of waste or residues and dedicated feedstocks. However, the average land-use intensity of residue and dedicated biomass differs by four orders of magnitude. To represent an upper boundary on our results, we could assume all biomass comes from dedicated feedstock production. This upper bound estimate results in biomass comprising over 99% of the total land use in future energy scenarios (unless the scenario excludes biomass). The GP, WWF, and GEA energy scenarios reviewed here specify that the biomass in their scenarios should come only from forestry and agricultural wastes and residues, rather than dedicated production. The level of biomass required in those scenarios is within the range of global technical potential [53], but estimates of global technical potential do not reflect economic or geographic constraints on biomass residue recovery (see Supplementary Figure S3). There is also evidence at the regional level that residues alone are unlikely to meet bioenergy demand, which could result in increased logging and displacement of other wood products [55]. To take a lower bound on biomass, we could assume all feedstock comes from waste or residue. Then biomass constitutes only about 1% of total land use in future energy scenarios.


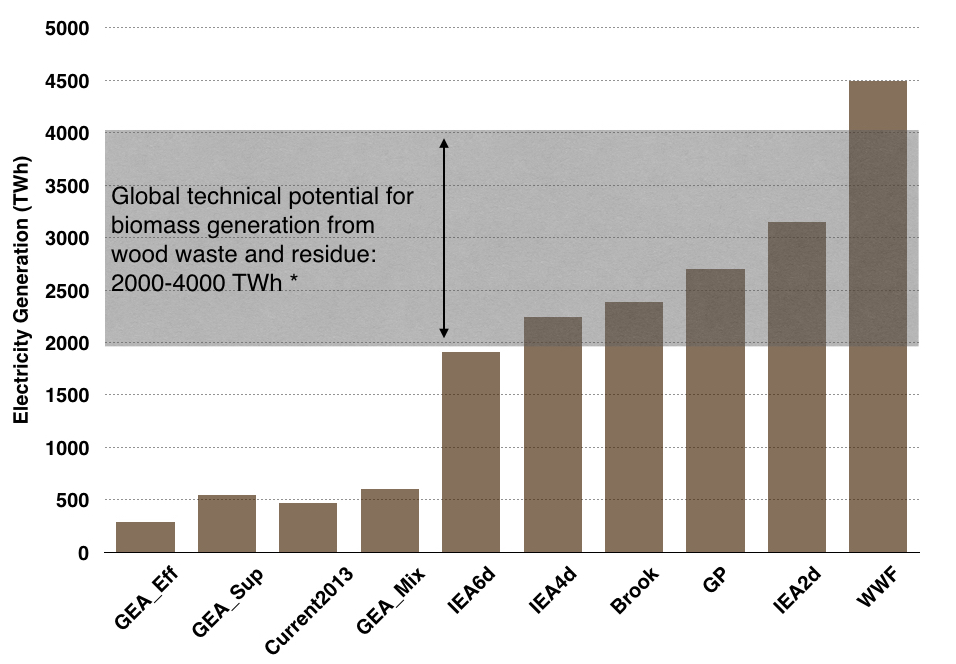


**Supplementary Figure S3.** Amount of electricity sourced from biomass in each of the scenarios we evaluated. Most scenarios do not specify whether the biomass will be sourced from dedicated crops or managed forests, or sourced from waste and residue. However, several scenarios include more biomass combustion than could be reasonably sourced from waste and residues, assuming all waste and residue produced globally could be economically collected. *Global technical potential for biomass production comes from Searchinger and Heimlich (2015).

**Supplementary Information References**

[1] Fthenakis V, Kim HC. Land use and electricity generation: A life-cycle analysis. *Renew Sustain Energy Rev* 2009; 13: 1465–1474.

[2] Hertwich EG, Gibon T, Bouman E a., et al. Integrated life-cycle assessment of electricity-supply scenarios confirms global environmental benefit of low-carbon technologies. *Proc Natl Acad Sci* 2015; 112: 6277–6282.

[3] Spitzley D V, Keoleian GA. *Life Cycle Environmental and Economic Assessment of Willow Biomass Electricity: A Comparison with Other Renewable and Non-Renewable Sources*. 2005.

[4] Jacobson MZ. Review of solutions to global warming, air pollution, and energy security. *Energy Environ Sci* 2009; 2: 148.

[5] Smil V. *Power Density Primer : Understanding the Spatial Dimension of the Unfolding Transition to Renewable Electricity Generation ( Part I – Definitions )*. 2010.

[6] Gates DM. *Energy and Ecology*. Sunderland, MA: Sinauer Associates Inc, 1985.

[7] Jordaan SM. *The land use footprint of energy extraction in Alberta*. University of Calgary, 2010.

[8] McDonald RI, Fargione J, Kiesecker J, et al. Energy sprawl or energy efficiency: climate policy impacts on natural habitat for the United States of America. *PLoS One* 2009; 4: e6802.

[9] DOE. *Energy technology characterizations handbook: environmental pollution and control factors*, http://hdl.handle.net/2027/uc1.b4236598 (1983).

[10] Skone TJ, Littlefield J, Marriott J, et al. *Life Cycle Analysis of Natural Gas Extraction and Power Generation*. 2014.

[11] Bryce R. *Power Hungry: The Myths of ‘Green’ Energy and the Real Fuels of the Future*. New York: PublicAffairs, 2011.

[12] Copeland HE, Pocewicz A, Kiesecker JM. *Geography of energy development in Western North America: potential impacts on terrestrial ecosystems*. 2011.

[13] OECD/NEA. *Uranium 2014: Resources, Production and Demand*, http://www.oecd-nea.org/ndd/pubs/2012/7059-uranium-2011.pdf (2014).

[14] US Nuclear Regulatory Commission. Generic Environmental Impact Statement for License Renewal of Nuclear Plants (NUREG-1437 Vol. 1), http://www.nrc.gov/reading-rm/doc-collections/nuregs/staff/sr1437/v1/part02.html (2012, accessed 20 July 2016).

[15] Schneider E, Carlsen B, Tavrides E, et al. A Top Down Assessment of Energy, Water and Land use in Uranium Mining, Milling, and Refining. *Energy Econ*. Epub ahead of print September 2013. DOI: 10.1016/j.eneco.2013.08.006.

[16] Finch WI. Uranium, Its Impact on the National and Global Energy Mix. *USGS*.

[17] Eliasson B, Lee Y. *Integrated Assessment of Sustainable Energy Systems in China*. Dordrecht, Netherlands: Kluwer Academic Publishers, 2003.

[18] Harries J, Levins D, Ring B, et al. Management of waste from uranium mining and milling in Australia. *Nucl Eng Des* 1997; 176: 15–21.

[19] Rossing Uranium Limited. *Report to stakeholders 2015: Moving ahead, realising our vision.* 2015.

[20] About BB, November T, Jacobson MZ, et al. BraveNewClimate Critique of ‘ A path to sustainable energy by 2030 ′. 2009; 1–83.

[21] Bondarkov MD, Oskolkov BY, Gaschak SP, et al. Environmental Radiation Monitoring in the Chernobyl Exclusion Zone—History and Results 25 Years After. *Health Phys* 2011; 101: 442–485.

[22] World Nuclear Association. Fukushima Accident, http://www.world-nuclear.org/information-library/safety-and-security/safety-of-plants/fukushima-accident.aspx (2016, accessed 7 July 2016).

[23] BP. *BP statistical review of world energy*, http://www.bp.com/en/global/corporate/energy-economics/statistical-review-of-world-energy.html (2016, accessed 31 July 2014).

[24] Deryabina TG, Kuchmel S V., Nagorskaya LL, et al. Long-term census data reveal abundant wildlife populations at Chernobyl. *Curr Biol* 2015; 25: R811–R826.

[25] International Commission on Large Dams. World Register of Dams, www.icold-cigb.net/GB/World_register/world_register.asp (2015, accessed 27 August 2015).

[26] International Energy Agency (IEA). *Technology Roadmap: Hydropower*. Epub ahead of print 2012. DOI: 10.1007/SpringerReference_7300.

[27] Dijkman TJ, Benders RMJ. Comparison of renewable fuels based on their land use using energy densities. *Renew Sustain Energy Rev* 2010; 14: 3148–3155.

[28] Kumar A, Cameron JB, Flynn PC. Biomass power cost and optimum plant size in western Canada. *Biomass and Bioenergy* 2003; 24: 445–464.

[29] Smil V. *Power Density*. Cambridge, MA: MIT Press, 2015.

[30] EIA 860.

[31] EIA 923.

[32] Hernandez RR, Hoffacker MK, Murphy-Mariscal ML, et al. Solar energy development impacts on land-cover change. *Proc Natl Acad Sci* 2015; 112: 13579–13584.

[33] Ong S, Campbell C, Denholm P, et al. *Land-Use Requirements for Solar Power Plants in the United States*. 2013.

[34] Solar Energy Industries Association. Major Solar Projects in the United States Operating, Under Construction, or Under Development, www.seia.org/research-resources/major-solar-projects-list (2015, accessed 5 October 2015).

[35] National Renewable Energy Corporation. Project List, http://narenco.com/projects/projects (2016, accessed 19 February 2016).

[36] US Bureau of Land Management. BLM California Solar Applications, www.blm.gov/ca/st/en/prog/energy/pendingapps.html (2015, accessed 19 April 2015).

[37] Bertani R. World geothermal power generation in the period 2001–2005. *Geothermics* 2005; 34: 651–690.

[38] Global Energy Observatory. Power Plant Online Database, http://globalenergyobservatory.org (2015, accessed 24 November 2015).

[39] BP. *BP statistical review of world energy*, https://www.bp.com/en/global/corporate/energy-economics/statistical-review-of-world-energy.html (2018).

[40] International Energy Agency. *Energy Technology Perspectives 2015*. 2015.

[41] Greenpeace, Global Wind Energy Council, European Renewable Energy Council. *Energy [R]evolution: A Sustainable World Energy Outlook*, http://www.greenpeace.org/international/en/publications/Campaign-reports/Climate-Reports/Energy-Revolution-2012/ (2012).

[42] World Wildlife Fund. *The Energy Report: 100% Renewable Energy by 2050*. 2011.

[43] International Institute for Applied Systems Analysis. *Global Energy Assessment: Toward a Sustainable Future*. Cambridge University Press. Epub ahead of print 2012. DOI: 10.1017/CBO9780511793677.

[44] Jacobson MZ, Delucchi MA. Providing all global energy with wind, water, and solar power, Part I: Technologies, energy resources, quantities and areas of infrastructure, and materials. *Energy Policy* 2011; 39: 1154–1169.

[45] Brook BW. Could nuclear fission energy, etc., solve the greenhouse problem? The affirmative case. *Energy Policy* 2012; 42: 4–8.

[46] Angel S, Parent J, Civco DL, et al. The dimensions of global urban expansion: Estimates and projections for all countries, 2000–2050. *Prog Plann* 2011; 75: 53–107.

[47] Smith P, Gregory PJ, van Vuuren D, et al. Competition for land. *Philos Trans R Soc Lond B Biol Sci* 2010; 365: 2941–57.

[48] Jacobson MZ, Delucchi MA, Bauer ZAF, et al. 100% Clean and Renewable Wind, Water, and Sunlight All-Sector Energy Roadmaps for 139 Countries of the World. *Joule* 2017; 1: 108–121.

[49] Trainor AM, McDonald RI, Fargione J. Energy Sprawl is the Largest Driver of Land Use Change in United States. *PLoS One* 2016; 11: 1–16.

[50] Barros N, Cole JJ, Tranvik LJ, et al. Carbon emission from hydroelectric reservoirs linked to reservoir age and latitude. *Nat Geosci* 2011; 4: 593–596.

[51] de Faria FAM, Jaramillo P, Sawakuchi HO, et al. Estimating greenhouse gas emissions from future Amazonian hydroelectric reservoirs. *Environ Res Lett* 2015; 10: 124019.

[52] Evans A, Strezov V, Evans TJ. Sustainability considerations for electricity generation from biomass. *Renew Sustain Energy Rev* 2010; 14: 1419–1427.

[53] Searchinger T, Heimlich R. *Avoiding Bioenergy Competition for Food Crops and Land*. 2015.

[54] Sanchez DL, Nelson JH, Johnston J, et al. SUPPLEMENTARY INFO: Biomass enables the transition to a carbon-negative power system across western North America. *Nat Clim Chang* 2015; 3–7.

[55] Abt RC, Abt KL, Cubbage FW, et al. Effect of policy-based bioenergy demand on southern timber markets: A case study of North Carolina. *Biomass and Bioenergy* 2010; 34: 1679–1686.
