# Supplementary material for: Land-use intensity of electricity production and tomorrow’s energy landscape
Source: PLoS One. 2022 Jul 6;17(7):e0270155. doi: 10.1371/journal.pone.0270155 (PMC9258890; doi:10.1371/journal.pone.0270155)
Supplement: S1 Table — (DOCX) [file pone.0270155.s002.docx]

Table S1. For each fuel source, we list the sources of land use data that we included in our analysis. Data sources generally included both land use and electricity production for specific sites, unless otherwise noted.

| Generation Fuel/Technology | Data Source | Direct or Indirect | Specifics | Geographic Range |
| --- | --- | --- | --- | --- |
| Coal | Fthenakis & Kim (2009) | Direct & Indirect | Real power plant and mining data for 13 sites in the US | U.S., covers all coal-mining regions |
|  | Gates, David M (1985) | Direct & Indirect | One underground and one surface mining location | U.S. |
|  | Hertwich et al. (2014) | Direct & Indirect | Three combustion technologies for generic US hard coal mining with rail transport | U.S. |
|  | Jordaan (2010) | Indirect | Survey of Alberta coal mines | Alberta, Canada |
|  | Spitzley & Keoleian (2005) | Indirect | Indirect based on Illinois surface coal mine | Illinois, U.S. |
|  | McDonald et al. (2009) | Indirect | Least compact mine based on single site in U.S. Most compact taken from Spitzley & Keoleian (2005) | U.S. |
|  | Smil (2010) | Direct & Indirect | Single plant & associated mine: the Robert Scherer Plant in Georgia | Georgia, U.S. |
| Natural Gas | Boyce & Naugle (2011) | Indirect | Geospatial analysis of western US, includes whole area of production field | Western U.S. |
|  | Spitzley & Keoleian (2005) | Direct | Power plant and pipeline area | U.S. |
|  | Smil (2010) | Direct | Three turbine technologies | Generic technology |
|  | Jordaan (2010) | Indirect | Geospatial analysis of wells | Alberta, Canada |
|  | Bryce (2011) | Indirect | Well pad only | US |
|  | Jordaan (2017) | Direct & Indirect | Five sites including ﻿production, gathering, processing, transmission and power generation | Texas, U.S. (Barnett Shale) |
|  | DOE (1983) | Direct & Indirect | Includes extraction, purification, pipeline transport, storage, and power plant area. | U.S. |
|  | McDonald et al. (2008) | Indirect | Extraction and pipelines | U.S. |
|  | Copeland (2011) | Indirect | Whole extraction area (spacing) | Western U.S. |
|  | NETL (2014) | Direct & Indirect | Different combustion technology, different extraction formations (conventional and unconventional: shale gas, tight gas) | U.S. |

Supplementary Table S1. Continued…

| Generation Fuel/Technology | Data Source | Direct or Indirect | Specifics | Geographic Range |
| --- | --- | --- | --- | --- |
| Biomass | Dijkman & Benders (2010) | Indirect | European yield averages: high, medium, low yields for willow and poplar | Europe |
|  | Fthenakis & Kim (2009) | Indirect & Direct | Poplar yields, with three different combustion technologies | U.S. |
|  | Kumar (2003) | Indirect | Hardwood and Spruce Yields | Alberta, Canada |
|  | McDonald et al. (2009) | Indirect | Willow gasification | U.S. |
|  | Smil (2010) | Indirect & Direct | Poplar and Willow in temperate climate | U.S. |
|  | Spitzley & Keoleian (2005) |  | Poplar in Midwestern US, Willow in New York, with three different processing technologies | U.S. – Midwest and New York state |
| Geothermal | Bertani (2005) | Direct | Electricity generation data from Bertani (2005), combined with original geospatial measurement | Across 11 countries on six continents |
| Hydropower | International Commission on Large Dams (ICOLD) Dataset | Direct | 951 dams that were classified as single use, i.e. only used for hydroelectric generation. Data on reservoir area and annual electricity generation | Hydroelectric dams in 57 countries across 6 continents |
| Solar PV | Hernandez et al. (2015) | Direct | Public documents for area, EIA data for electricity | California |
|  | Original Data Collection | Direct | Public documents for area, EIA data for electricity | Across four U.S. states |
|  | Ong et al. (2013) | Direct | Public documents and satellite imagery for area, EIA data for electricity. | Across 14 U.S. states |
| Solar CSP | Hernandez et al. (2015) | Direct | Public documents for area, EIA data for electricity, two plants in CA | California, U.S. |
|  | Ong et al. (2013) | Direct | Twenty plants across 5 US states: CA, AZ, FL, NV, CO | Across 5 U.S. States |
|  | Lilliestam et al. (2021)  Piemonte et al. (2011)  Smil (2015) | Direct  Direct  Direct | 76 sites w/ estimated generation  Case study of one plant  Case studies of two sites | Across 13 countries on 5 continents  Italy  Spain |
| Wind Turbines | Original Data Collection | Direct | Geospatial measurement of land area, annual electricity generation data from EIA. 58 wind farms | Across 23 U.S. states |

Supplementary Table S1. Continued…

| Generation Fuel/Technology | Data Source | Direct or Indirect | Specifics | Geographic Range |
| --- | --- | --- | --- | --- |
| Nuclear | Original Data Collection | Direct | Geospatial measurement of 59 nuclear sites: power plant area, including cooling water and on-site waste storage. | 31 U.S. states |
|  | Fthenakis & Kim (2009) | Indirect | Full fuel cycle, including waste | U.S. |
|  | Finch (1997) | Indirect | Mining and milling, open pit | U.S. |
|  | Eliasson & Lee (2003) | Indirect | Mining and milling, open pit and underground mines | China |
|  | Harries et al. (1997) | Indirect | Mining and milling, open pit and underground | Australia |
|  | Jacobson (2009) | Indirect | Mining and waste disposal (50% open pit, 50% underground) | U.S. |
|  | OECD (2008) | Indirect | Mining only, underground mine | Canada |
|  | OSTI | Indirect | Just mining. Low, Medium, and High estimate for three types of mining: Deep, Surface, and Solution | U.S. |
|  | Rossing (2010) | Indirect | Mining and milling, open pit | Namibia |
