# Supplementary material for: Land-use intensity of electricity production and tomorrow’s energy landscape
Source: PLoS One. 2022 Jul 6;17(7):e0270155. doi: 10.1371/journal.pone.0270155 (PMC9258890; doi:10.1371/journal.pone.0270155)
Supplement: Attachment — Submitted filename: Response to Reviewers.docx [file pone.0270155.s004.docx]

Dear Reviewers,

Thank you for taking the time to read our manuscript and providing your thoughtful comments. We have responded to all of your comments and believe they have improved the quality and clarity of the manuscript.

Thank you,

Jessica Lovering and co-authors

| **Reviewer #1** | |
| --- | --- |
| In the manuscript, the specific method for calculating coal indirect land area is unclear. | Thank you. We apologize for this oversight and appreciate you identifying this gap as it is really important for us to be explicit about our methodologies. We have edited the text to make it clearer. The new text reads:  “Indirect land use, specifically from mining, is the dominant contributor to coal’s land-use intensity, and thus a broader survey that focuses on land occupation for regional coal mining can give a more comprehensive estimate. Jordaan (2010) and McDonald et al. (2009) perform surveys of the land used for coal mining in Canada and the US, respectively [13, 31]. These two studies of indirect land-use were given in units of embodied energy for the mined coal, so we applied a 35% conversion efficiency to convert to electricity units, based on Ftheankis & Kim (2009). This gives an estimate of hectares per TWh for just the indirect, mining component of coal’s land use intensity.” |
| The study uses a large amount of data from multiple countries and states in the United States, which undoubtedly increases the authority of the manuscript. However, are the data collected within a similar time period, e.g. recent 5 years or 10 years? | Wherever possible, we sought and used the most recent data available. That said, we aggregated datasets from many different published studies and thus the time periods are all unique, depending on when they performed their surveys or case studies. Table 1 in the Supplementary Information provides a list of where data came from for each electricity source, and the date the study was performed. |
| The results of this study suggest that the IEA 6 Degree scenario has the smallest land-use area, which is a business-as-usual scenario. However, the use of a large amount of fossil energy will bring environmental problems. Can the environmental effects be taken into account to calculate and compare the generalized LUIE for various future scenarios, for example, converting the economic loss per unit of carbon emissions into an equivalent increase in land-use area? | One goal of this work was to expand the metrics available (i.e., describing land-energy relationships) to evaluate the environmental impacts of the energy system, rather than convert everything to a single metric (as is often done with energy systems using solely GHG emissions). While we included a graph comparing LUIE to GHG emissions for each energy source, the same could also be done for energy scenarios as you astutely suggest! This is an exciting opportunity to produce additional results from this current manuscript but we believe this is beyond the scope of the current manuscript and that it requires a separate, exhaustive analysis that would exceed the space limitations and scope of the existing manuscript. .  Specifically, such work would require the development of assumptions that would require significantly more research to establish and refine.That said, we sincerely appreciate the excellent idea and would be keen to pursue this at a later time. |
| The calculation results of LUIE of different studies for the same power source are quite different. This may be attributed to the fact that the amount and weight of data used in each study are different or different calculation standards are used. Using a large amount of data can enhance the accuracy of the calculation results. But how to explain the rationality of the calculation standards chosen in the manuscript? | On the contrary, a conclusion of this study is that there just is a lot of variation in land use within a given generating technology, which is consistent with previous studies. That is why previous studies that relied on single case studies (or a small number of power plants) were so problematic. We do appreciate you bringing this to light as we believe we can do a better job of providing additional context on this issue. To that end, we have added this statement:  “We anticipate large variability in results within and among energy technologies, as has been demonstrated and discussed by previous studies (MacKay 2013, Capellán-Pérez et al. 2017 , Jordaan et al. 2017, and Hernandez et al. 2022)”    We used a standard and simple methodology for calculating land use across energy technologies that includes measuring occupied land and dividing by annual electricity generation. It’s quite consistent across studies. The cause of variation is much more likely to be geographical, technological, or based on fuel quality. |
| **Reviewer #2** | |
| I do have one concern which I don’t consider trivial and that is with respect to the storage of spent nuclear fuel. It is true that currently nuclear fuel in the United States is stored onsite. However our stockpile of spent fuel is growing rapidly. For example, An April 2020 report from the Congressional Research Service indicates that the amount of spent nuclear fuel in storage is expected to double by 2048. This is an issue which is unique to nuclear because unlike a hydroelectric dam or natural gas power plant which has a more-or-less fixed footprint at construction, the footprint associated with a nuclear powerplant will continue to grow throughout its lifecycle. I think that if the authors acknowledge this and work it into their paper it likely won’t alter the fundamental findings of the paper and would go a long way to strengthening the paper. | Thank you for highlighting this important point. We actually found two studies that estimated land use intensity for spent fuel storage for the U.S., and they are included in Table 1 in the SI, but we failed to include them in the main manuscript or SI text. That has now been corrected.  You are correct that the magnitude of LUIE for spent fuel storage is very small, but it is still important to discuss this unique impact from nuclear. |
| For question 3 – “Have the authors made all data underlying the findings in their manuscript fully available?” I’m not entirely sure how to address this. The compiled data set was generated entirely from publicly available information and the authors do cite each source from which data were obtained. However, the authors do not provide the compiled data set (or, at the very least, I wasn’t able to access it). | Our apologies, we made our LUIE data available as a spreadsheet in the Supplementary Information, but perhaps it was not easy to access? We will make sure it is available when published. |
| **Reviewer #3** | |
| According to the principle of thermodynamics, I am not 100% sure you can write "energy production" or similar. Better to write "energy conversion or management"? | Yes, this is a very good point. This paper focuses on electricity, which is generated. We have gone through and made sure the language is accurate in the manuscript. |
| You wrote "To provide LUIE results representative of the current state of each energy technology, we required that data sources represent existing, operational energy facilities and real world, rather than modeled, electricity generation data". What you precisely mean? Did you get the time series data or you got for instance the annual power production for those specific plants from literature (i.e. TWh/year/plant)? | We acquired annual electricity generation for individual power plants for the most recent year available. We also included data from studies that did the same. This is in contrast to some rougher estimates of land use intensity that model electricity generation based on nameplate capacity and a generic capacity factor.  We have made several small edits throughout the manuscript to help clarify. |
| What could be the effect of climate and technological advancements (i.e. increased efficiencies) on your analysis? | This is a great question and an interesting thought experiment. We have added the following sentence:  “Similarly, technological advances that improve efficiency and/or electricity generation could arguably reduce LUIE but evidence of a causal relationship is lacking. For example, the manufacturing and construction of bifacial PV (light collected from both sides of the panel surface) is driven by estimates of 20-40% electricity gains over monofacial panels, which could ostensibly drive smaller power plant footprints to meet finite electricity needs. However, exponential increases in electricity demands globally and the nascent state of the technology’s economic and reliability outcomes mean that impacts on real-world LUIE are yet to be determined (Khan et al. 2021).” |
| Some energy sources, as you have pointed out depends on locations, for instance solar is driven by latitude, could you further discuss on this or even better plot them in a map? | When we compared LUIE for solar PV sites with their local solar insolation, we did not find a robust correlation. While this might be quite surprising, the explanation is that generation can depend much more on technology like tracking than on pure solar resources. The earliest renewables projects tended to be sited in places with the best resources, or economic potential, but as the technology has improved, renewables projects can be economic in more varied geographies.  We agree that a rapid, renewable energy transition requires not only understanding how much land will be required but where land will be transformed. While projecting where energy development may unfold is beyond the scope of the this manuscript, we have added the following sentence to point the reader to some of the available, spatially-explicit models that do seek to address this question:  “Studies seeking to map existing and future energy infrastructure (e.g., Jenkins et al., 2021) may, in part, inform decisions regarding impacts on wildlife; however, projections including spatially-explicit footprints require robust, accurate, and representative model parameters.” |
| Can you discuss about other emerging technologies? and or provide further suggestions on how to avoid this energy and land use conflict (i.e., marine energy, agrivoltaics, supercritical geothermal, etc.)? | We have made edits to this section to provide examples of emerging technologies and suggestions on how to avoid land-related trade-offs with food and conservation:  “Some power technologies can produce electricity without requiring additional land. Solar PV can be placed on rooftops, over parking lots, or on top of inland water body surfaces, known as floatovoltaics (Cagle et al. 2020) ; PV and wind turbines can be built on degraded, contaminated, or on top of agricultural land—the latter with PV, known as an agrivoltaic systems (Hernandez et al. 2019) ; biomass feedstock can be sourced from residues and waste materials [64]; and nuclear power plants (as well as wind and PV) can be built offshore (Vo et al. 2021, Guo et al. 2022) . Dams that were originally constructed for water supply, irrigation, or flood control can be retrofitted with hydroelectric capabilities [70].” |
